# A component-based system for agricultural drought monitoring by remote sensing

**DOI:** 10.1371/journal.pone.0188687

**Published:** 2017-12-13

**Authors:** Heng Dong, Jun Li, Yanbin Yuan, Lin You, Chao Chen

**Affiliations:** 1 School of Resources and Environmental Engineering, Wuhan University of Technology, Wuhan, Hubei, China; 2 College of Geoscience and Surveying Engineering, China University of Mining and Technology, Beijing, China; 3 Institute of Remote Sensing and Geographic Information Systems, Peking University, Beijing, China; 4 Marine Science and Technology College, Zhejiang Ocean University, Zhoushan, Zhejiang, China; Institute for Sustainable Plant Protection, C.N.R., ITALY

## Abstract

In recent decades, various kinds of remote sensing-based drought indexes have been proposed and widely used in the field of drought monitoring. However, the drought-related software and platform development lag behind the theoretical research. The current drought monitoring systems focus mainly on information management and publishing, and cannot implement professional drought monitoring or parameter inversion modelling, especially the models based on multi-dimensional feature space. In view of the above problems, this paper aims at fixing this gap with a component-based system named RSDMS to facilitate the application of drought monitoring by remote sensing. The system is designed and developed based on Component Object Model (COM) to ensure the flexibility and extendibility of modules. RSDMS realizes general image-related functions such as data management, image display, spatial reference management, image processing and analysis, and further provides drought monitoring and evaluation functions based on internal and external models. Finally, China’s Ningxia region is selected as the study area to validate the performance of RSDMS. The experimental results show that RSDMS provide an efficient and scalable support to agricultural drought monitoring.

## 1. Introduction

Drought is one of the most common natural disasters, and often causes significant environmental, agricultural, healthy, economic and social consequences, especially in developing economies [1–3]. As for scientists and researchers, detecting the occurrence and severity of the drought disaster by observing the spatio-temporal changes of nature is an important challenge [4,5]. In this field, meteorologists, hydrologists, geophysicists proposed many methods and models from different perspectives. Su et al. [6] summarized them into three categories: meteorological indexes (e.g., the Standardized Precipitation Index), process-based indexes (e.g., evaporative fraction) and satellite-based indexes (e.g., vegetation indexes). All of the above indexes are composed of geophysical parameters, e.g. land surface temperature, soil moisture, vegetation water content, surface albedo, etc. Therefore, the measurement of geophysical parameters largely determine the precision and accuracy of monitoring.

Traditional measurements of geophysical parameters are point-based, which mainly rely on fixed or mobile stations [7–11]. The interpolation methods and the representativeness of the observation points have been discussed a lot to extend the point-based observations to continuous data [12]. The rapid development of remote sensing technology in recent decades provides an effective solution to this problem [13–19], and the satellite-based drought indexes have attracted scholars’ attentions. Many effective models were proposed based on different ranges of electromagnetic spectrum to accommodate various kinds of underlying surface conditions. For instance, the thermal inertia model and the active microwave remote sensing model [20] were designed to monitor the drought over bare soils. For vegetated surfaces, the Crop Water Stress Index [21,22], the Temperature Vegetation Dryness Index [23], the Perpendicular Drought Index [24], the Shortwave Infrared Perpendicular Water Stress Index [25,26] have been widely used to monitor the drought status of the soil with different vegetation coverage. The continuous modification of the existing drought indexes and the continuous advent of new indexes are the inevitable trend of drought monitoring using remote sensing technology. Therefore, the drought monitoring systems which implement those indexes are becoming increasingly important.

The software platform is fundamental to drought monitoring services. The government agencies or organizations in many countries established drought monitoring platforms in succession, including US Drought Monitor (http://droughtmonitor.unl.edu), European Drought Observatory (edo.jrc.ec.europa.eu), Meteorological Drought Monitoring System of China (http://cmdp.ncc-cma.net/influ/dust.php), and African Flood and Drought Monitor (http://stream.princeton.edu/AWCM/WEBPAGE/interface.php) [27–32]. A few scholars and research teams also developed drought monitoring and publishing systems. Deng et al. [33] built an on-demand web service system named Global Agriculture Drought Monitoring and Forecasting System (GADMFS), which significantly improves global agriculture drought monitoring, prediction and analysis. Hao et al. [34]developed the Global Integrated Drought Monitoring and Prediction System (GIDMaPS), which provides meteorological and agricultural drought information based on multiple satellite-based and model-based precipitation and soil moisture datasets. Sheffield et al. [35]developed a drought monitoring and seasonal hydrological forecast system for sub-Saharan Africa contributes to building capacity through technology and knowledge transfer.

Although great progress has been made in drought monitoring technology, there are still work to do on the development of software and platforms. On one hand, the current drought-related platforms are often designed for a single purpose and focus mainly on information management and publishing, which cannot satisfy the advanced needs in research and professional work; On the other hand, the existing powerful remote sensing image processing software such as ENVI provide no specific module for drought monitoring, which makes a variety of complex and diverse inversion models cannot be well applied, especially the models based on multi-dimensional feature space. Furthermore, the architecture of most existing software does not allow users to incorporate new or customized models. The above limitations hinders the use of remote sensing technology in drought monitoring. Therefore, in the community of remote sensing, there is a growing need for a professional drought monitoring system with high flexibility and extendibility.

The objective of this paper is to design and develop RSDMS, a component-based remote sensing drought monitoring system. In RSDMS, the data management, image process, feature space analysis, drought monitoring and drought assessment are developed as independent and reusable components. All necessary procedures of drought monitoring can be performed conveniently, including radiometric calibration and atmospheric correction of remote sensing images, feature line extraction, parameter inversion etc., and new inversion models can be added into the system flexibly.

This paper introduces the overall system architecture, internal data structure and the module interface design (Section 2) followed by a detailed description of the development of all features (Section 3). The paper demonstrates the application of RSDMS in drought monitoring and assessment of China’s Ningxia region (Section 4) and discusses the characteristics of RSDMS and looks in to the future (Section 5).

## 2. Design and architecture

This section will introduce the overall architecture, internal data structure and interface design of RSDMS. RSDMS is designed and developed based on Component Object Model (COM) to ensure the flexibility and extendibility of common function modules and drought monitoring models. The component technology develops on the basis of the object-oriented technology to archive the software reuse.

### 2.1 Component Object Model

Component Object Model (COM) is a binary interface standard of software component designed by Microsoft Corporation in 1993. It is used to enable inter-process communication and dynamic object creation in a large range of programming languages. The essence of COM is a language-neutral way to implementing objects that can be used in environments different from the one in which they were created, even across machine boundaries. For well-authored components, COM allows reuse of objects with no knowledge of their internal implementation, as it forces component implementers to provide well-defined interfaces that are separated from the implementation.

The advantages of using COM are directly derived from the insertion or removal of the application, and the applications can evolve over time. In addition, there are some advantages of using COM to make the application more convenient and flexible, such as application customization, component library, and distributed components.

### 2.2 System workflow

**Fig 1:**
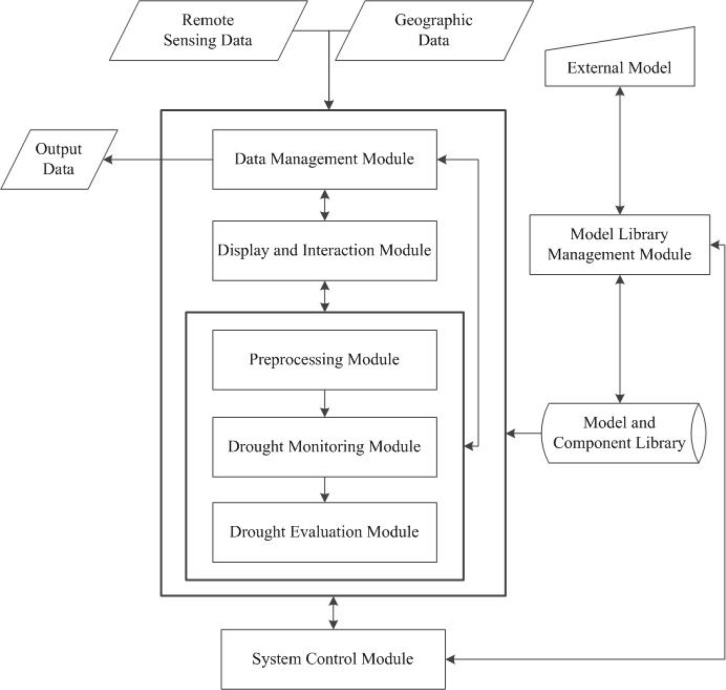
System workflow.

According to the conclusions of system demand analysis, this paper designs the function modules of the system as a whole, the system can be divided into data management module, display and interaction module, preprocessing module, drought monitoring module, drought evaluation module, system control module and model library management module. The system workflow of this paper as shown in Fig 1.

### 2.3 System architecture

**Fig 2:**
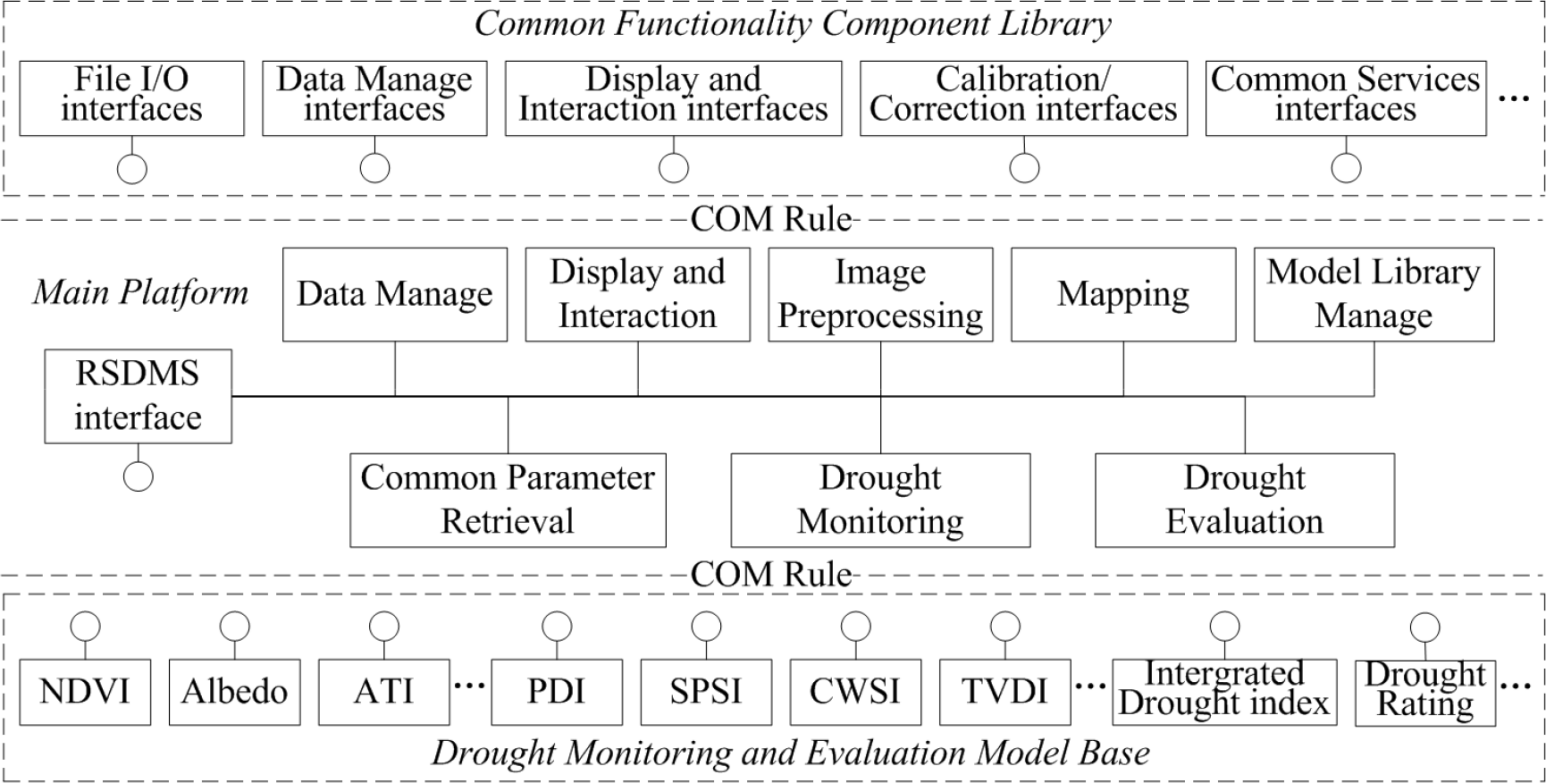
Architecture of RSDMS [36].

We design the architecture of RSDMS as shown in Fig 2, which is composed of three main parts: main platform, common functionality component library, and drought monitoring and evaluation model base.

#### 2.3.1 Main platform

Instructed by the design concept of COM, RSDMS is designed to be highly reusable and scalable. The main platform provides a friendly graphic user interface (GUI) as a container of function modules, and most functions are implemented by calling the corresponding component objects according to COM rules (The rectangle attached by a circle in Fig 1 represents an independent component). Besides, part of functions are encapsulated with the main platform to archive the reusability on the software-level, and they can be externally invoked in the form of a standard interface, namely “RSDMS interface”. On the basis of this interface, users can extend the functions by incorporating other functional modules, e.g. new data format input/output (I/O) drivers and new model components.

#### 2.3.2 Common functionality component library

The common functionality component library is the supporting infrastructure of RSDMS as a remote sensing platform. In this component library, the general image processing and display functions are grouped according to their correlations, and similar functions are encapsulated and developed as a component such as File I/O, data management (e.g. data subset, format conversions), display and user interaction, image preprocessing, and common service components as well (e.g. the progress reporting component). These modules can be flexibly assembled within RSDMS, and also be transportable across software.

#### 2.3.3 Drought monitoring and evaluation model base

This model base is the core part of RSDMS and supports the drought monitoring and evaluation service. It is composed of a range of geophysical parameter inversion models and drought-related index models. Each index model is encapsulated as an independent component abiding by a common interface regulation. As the main platform needs, the corresponding index model is called, then receives data from the main platform, and finally returns results back after model computation. In addition, a model management component is developed to operate the registration and management of external models.

### 2.4 Internal data structure

Vector and raster data are two major data formats in RSDMS; therefore, the internal data structures are designed for handling these two formats.

#### 2.4.1 Vector data

**Fig 3:**
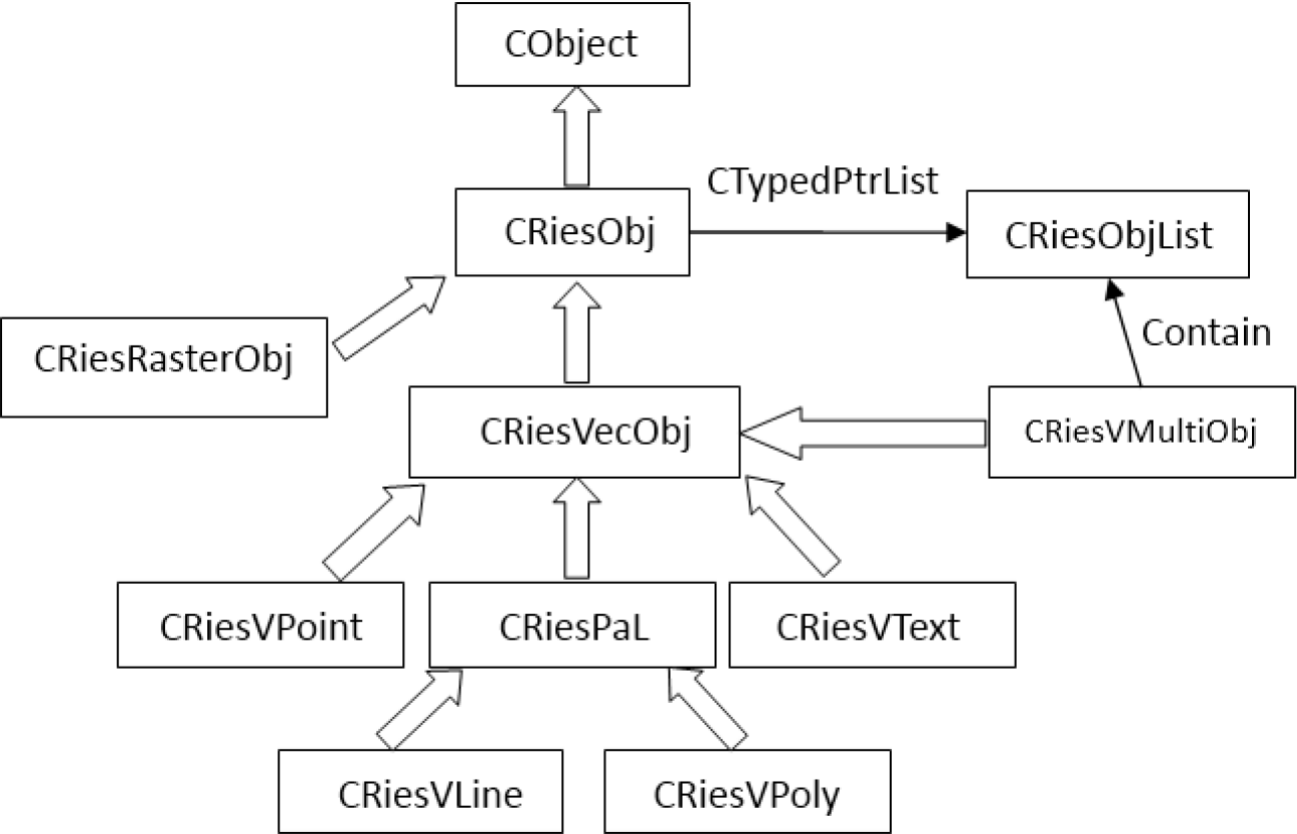
Vecotr data structure.

For vector data, we design vector objects based on traditional vector models (point, line, polygon and text annotation) as shown in Fig 3. Fig 3 shows the class inheritance relation of vector features. The basic class for geographic entities CRiesObj is defined based on a MFC class CObject, and the vector base class CRiesVecObj and the raster base class CRiesRasterObj inherits from CRiesObj. Furthermore, the point feature class CRiesVPoint, the line feature class CRiesVLine, the polygon feature class CRiesVPoly and the text feature class CRiesVText are defined based on CRiesVecObj. Apart from simple features, the multi-feature class CRiesVMultiObj is defined using the data type CTypedPtrList to handle possible multi-feature objects. The layer class CRiesLayer is defined as a container of vector features. The management of vector features is operated through corresponding feature classes, while their display control is implemented through the layer class.

#### 2.4.2 Raster data

**Fig 4:**
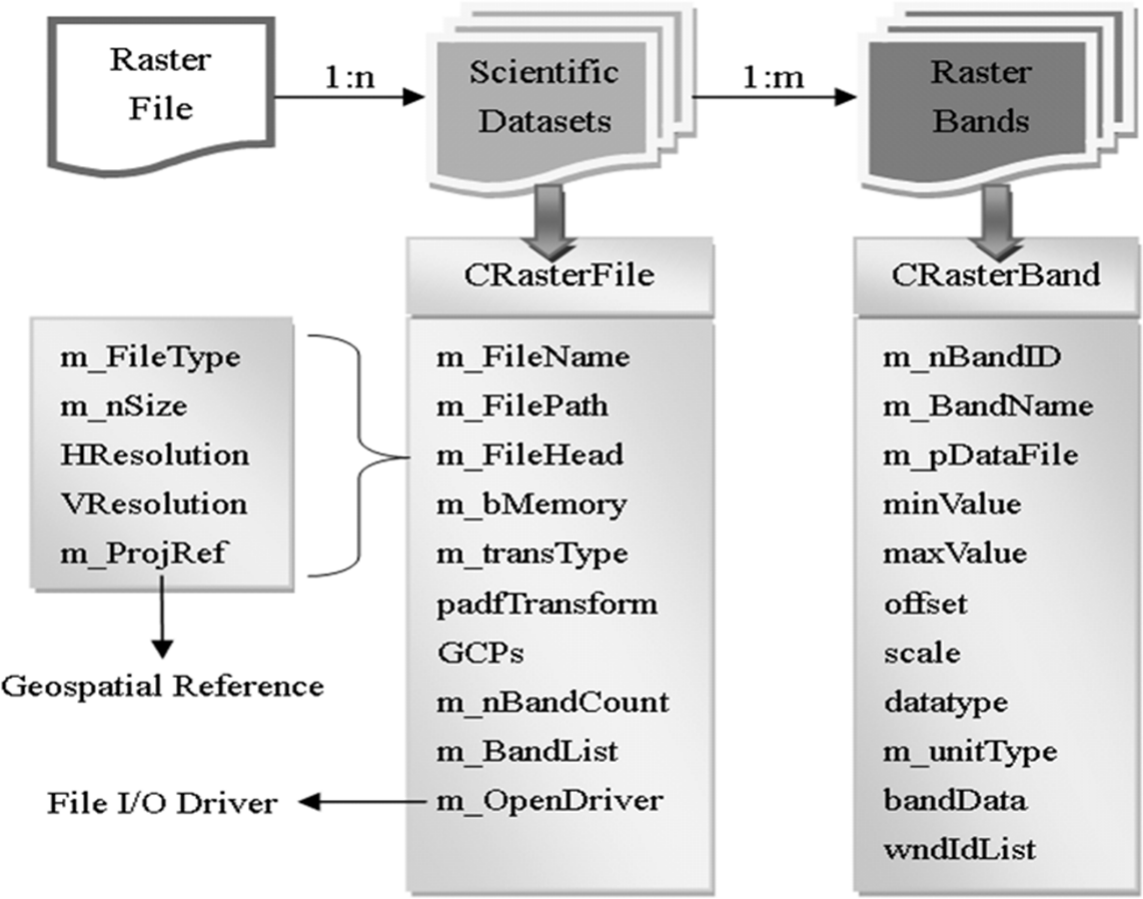
Raster data structure.

By analyzing the Geospatial Data Abstraction Library (GDAL) data model and other commonly used remote sensing data formats (ENVI, HDR, LD2 etc.), a simplified model for raster format is designed as shown in Fig 4. A raster file may contain multiple raster bands, especially the band number of hyperspectral images can reach to hundreds. Therefore, ***CRasterBand*** is defined to represent the raster bands, and the raster bands with the same properties (size, spatial resolution and spatial reference) are treated together as a Scientific Dataset (SD). In RSDMS, ***CRasterFile*** is used to store the SD, and the common properties are recorded in a structure—***m_Filehead***. Within the above class structures, a few member variables are defined to record the information about file, band etc. The raster data can be managed and operated flexibly on both band-level and file-level through the defined data structure.

### 2.5 Interface design

Fig 2 shows that the interaction between the main platform and function components is performed by interface access. In RSDMS, all function components except the display and interaction interface abide by common interface rules. The drought-related model components abide by the interface rule in Section 2.5.2, while the other components abide by the rule in Section 2.5.1.

#### 2.5.1 Interface design of main platform

Part of common functions are encapsulated with RSDMS, and they can be externally invoked as the interface “RSDMS Interface”. The modules inside or outside RSDMS can implement data I/O, output result etc. by invoking this interface. The detailed specification of this interface is shown in Table 1.

**Table 1:** Interface specification of the main platform.

Method name	Function	Return value	Remark
GetAppDirectory	Get the directory of the application	BSTR	None
GetBand	Get the pointer of the specified band	VOID	Para: VARIANT * *outVar* output the band pointer
GetBandData	Get the band data through a band pointer	VOID	Para: VARIANT * *bandVar* output the band data
GetLineParmManual	Get the parameters of the feature line obtained in a manual way	VOID	Return the slope and intercept of the feature line generated from the manual interaction with the 2D spectral feature space window
GetDataBlock	Get the specified data block of the specified band	VOID	Para: *bandVar* stores the pointer of the band to read, *yStart* and *yEnd* indicate the starting and ending row number of the data block respectively
WriteDataBlock	Write the specified data block of a specified band	VOID	Para: *bandVar* stores the pointer of the band to write, *yStart* and *yEnd* indicate the starting and ending row number of the data block respectively
CreateResultFile	Create a file for storing results	VOID	Create a file according to the specifications in the band or file templates VARIANT *tempBand and VARIANT *tempFile
BandsCompatibleTest	Test the compatibility of the band data	BOOL	Return the compatibility result
GetFileManual	Get one of files opened in the main platform	VOID	Para: VARIANT * *outVar*

#### 2.5.2 Interface design of model components

Compared with the complicated interface specification of the main platform, the interfaces of model components are relatively simplified. All model components can be performed by the four interface methods as shown in Table 2, which facilitates the unified management of all models in RSDMS.

**Table 2:** Interface specification of model components.

Method name	Function	Return value	Remark
Connect	Connect to the main platform, and receive the external interface pointer of the main platform	HRESULT	*lpUnknown*: the external interface pointer of the main platform
DisConnect	Disconnect with the main platform	HRESULT	Release the external interface pointer of the main platform
ProcessWithoutParm	Run model without input parameters	HRESULT	None
ProcessWithParm	Run model with input parameters	HRESULT	Parameters vary with models

The parameters, return value and function of the interface methods Connect, Disconnect and ProcessWithoutParm are fixed, while those for ProcessWithParm are customized according to the feature of models. The existence of ProcessWithParm can let the main platform or the other components perform a model component with some input parameters other than users’ interaction, and this mechanism is useful for the extension of model base.

## 3. Developments

### 3.1 Implementation

Considering that remote sensing image process and computation are involved in RSDMS, we choose C++ as the developing language since it is efficient and flexible for image processing. The Active Template Library and ActiveX are used to develop the components with and without graphic user interface respectively. Although most features are developed by the authors from scratch, some commonly used features are implemented based on open source libraries. The read and write of common raster data formats are realized by the Geospatial Data Abstraction Library (GDAL/OGR, http://www.gdal.org) under the open source license of Open Source Geospatial Foundation. The cartographic projection conversion are realized by the PROJ.4 library (https://github.com/OSGeo/proj.4/issues). Part of general image processing functions (e.g. histogram enhancement, spatial filtering) are implemented based on Intel’s OpenCV library (http://opencv.org). The matrix operations are implemented with the help of the CxImage library (http://www.codeproject.com/Articles/1300/CxImage).

### 3.2 System interface and features

**Fig 5:**
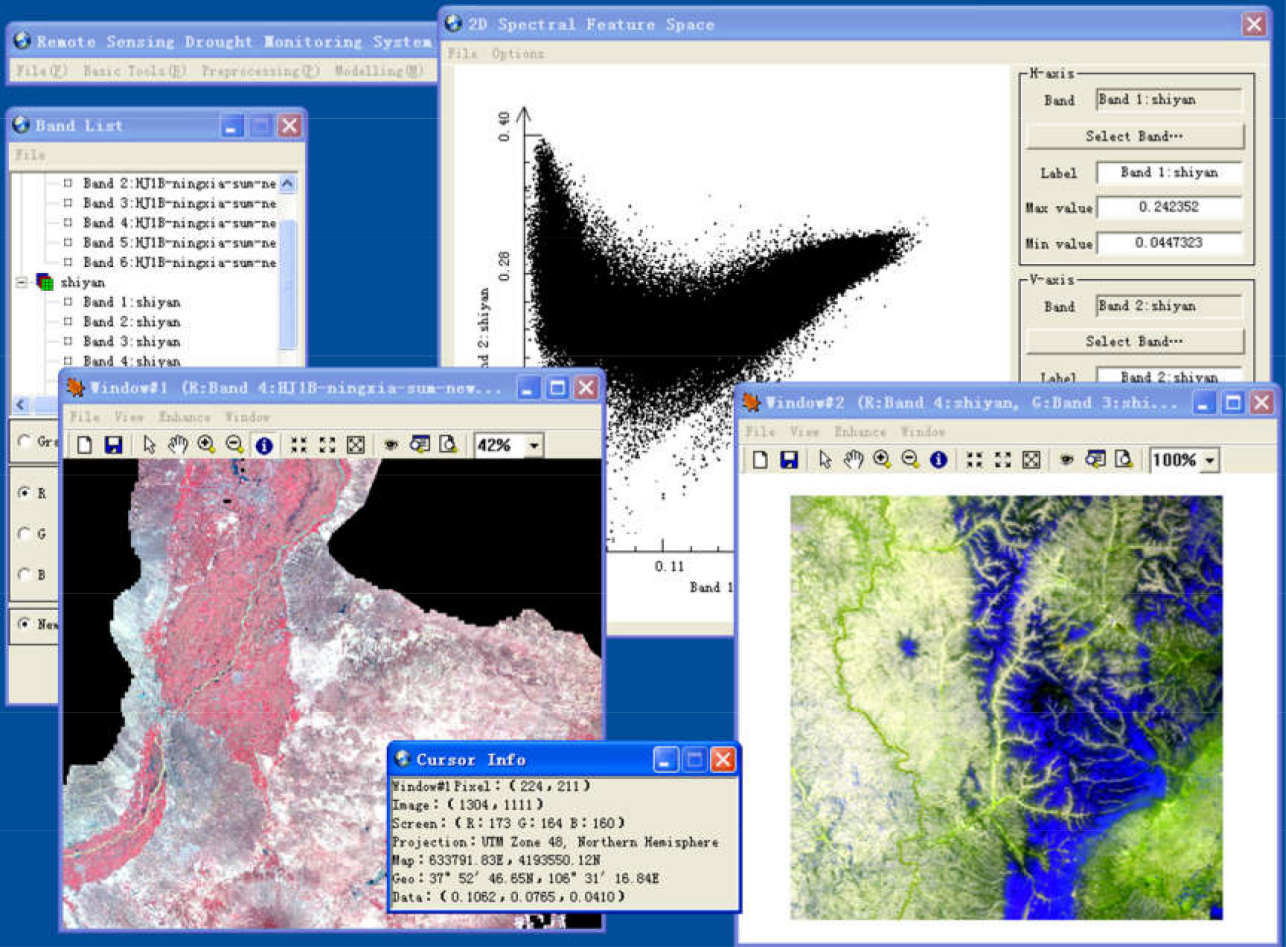
Main interface of RSDMS.

The main interface of RSDMS is shown in Fig 5. RSDMS provides two types of functions: as a general remote sensing platform, it provides a series of functions such as general image processing, analysis, display and data management; as a professional drought monitoring platform, it provides drought monitoring and evaluation function based on a bunch of inversion models.

#### 3.2.1 Data management

The support file formats of RSDMS are vector data, raster data and auxiliary data including ASCII and Binary format. The auxiliary data are mainly used to represent parameters of monitoring and evaluation models, and details can be referred to Section 3.2.4. The commonly used Shapefile format is selected to be the major vector format since it is suitable for data interexchange with other software. By contrast, the support raster data formats are more comprehensive because various kinds of satellite data or products are involved. Three kinds of raster data are supported in RSDMS: (1) the platform-specific formats of commercial remote sensing software, such as ENVI format, ERDAS IMAGINE format and so on; (2) satellite products such as the Moderate Resolution Imaging Spectroradiometer (MODIS) products in HDF format; (3) the specific data format used by government agencies, e.g. the Local File (LD2) format used by China’s meteorological departments.

Users often need to subset original images in the process of drought monitoring services, so RSDMS provides regular subset and subset via vector files. The former one is to subset images based on the input sample/line range or the top-left /bottom-right coordinates of images, while the latter one is based on the geographic extent of vector features. The Well-Known Text (WKT) defined by the Open Geospatial Consortium is used to represent geospatial projections involved in RSDMS. The projection file of Shapefile is directly read since it also adopts WKT to represent projection information, while for other file types such as ENVI format, HDF file and LD2, RSDMS extracts the projection and datum information according to the corresponding description file and transforms them into the WKT format.

#### 3.2.2 Display and information query

**Fig 6:**
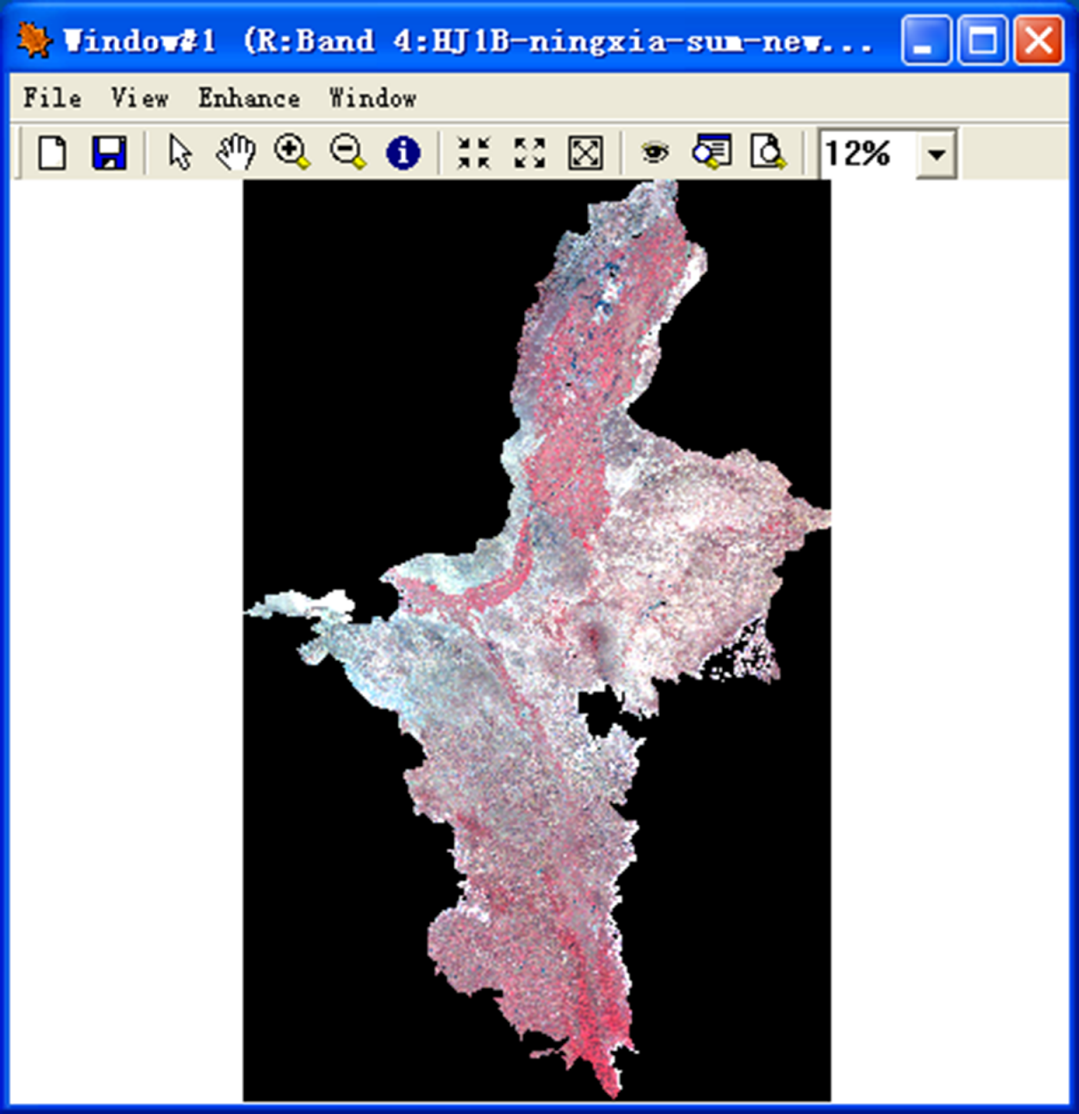
Image display and query.

The system handles image data display through image bands, and provides single band display and multi-band composition display (Fig 6). Twenty-eight color palettes are available for users to display a single band. Besides raster data, RSDMS can also show vector data and overlay display vector and raster data. It provides basic display functions e.g. zoom in/out, thumbnail image, and advanced functions such as brightness/contrast adjustment, filter enhancement. In addition, users can query the attribute information of vector data and the pixel information of raster data through a cursor locate tool.

#### 3.2.3 Preprocessing of remote sensing images

**Fig 7:**
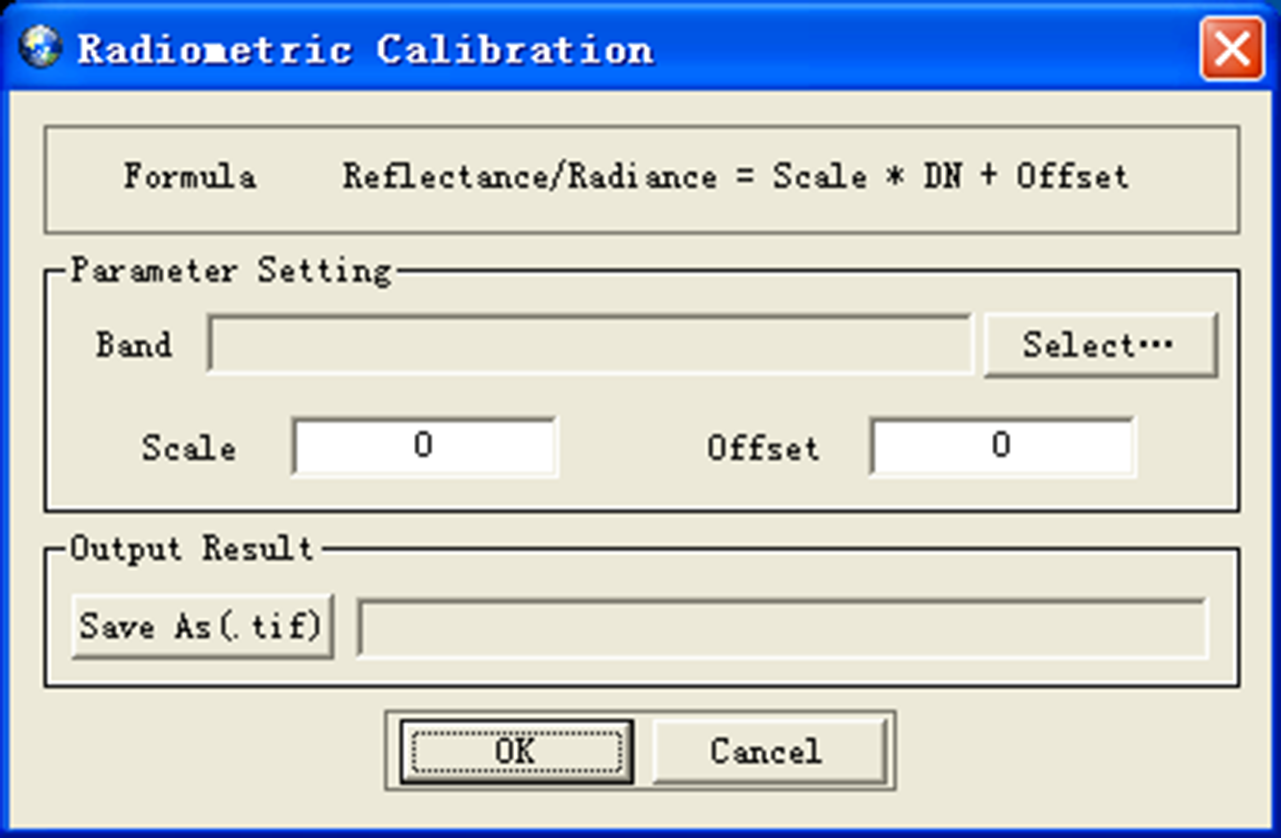
Radiometric calibration dialog.

RSDMS realizes the radiometric calibration using the equation introduced in [37]: Reflectance/Radiance = Scale * DN + Offset, where *Scale* and *Offset* are magnification times and correction coefficient for zero point, and *DN* is the digital number recorded by the image sensor. Fig 7 shows the radiometric calibration dialog.

**Fig 8:**
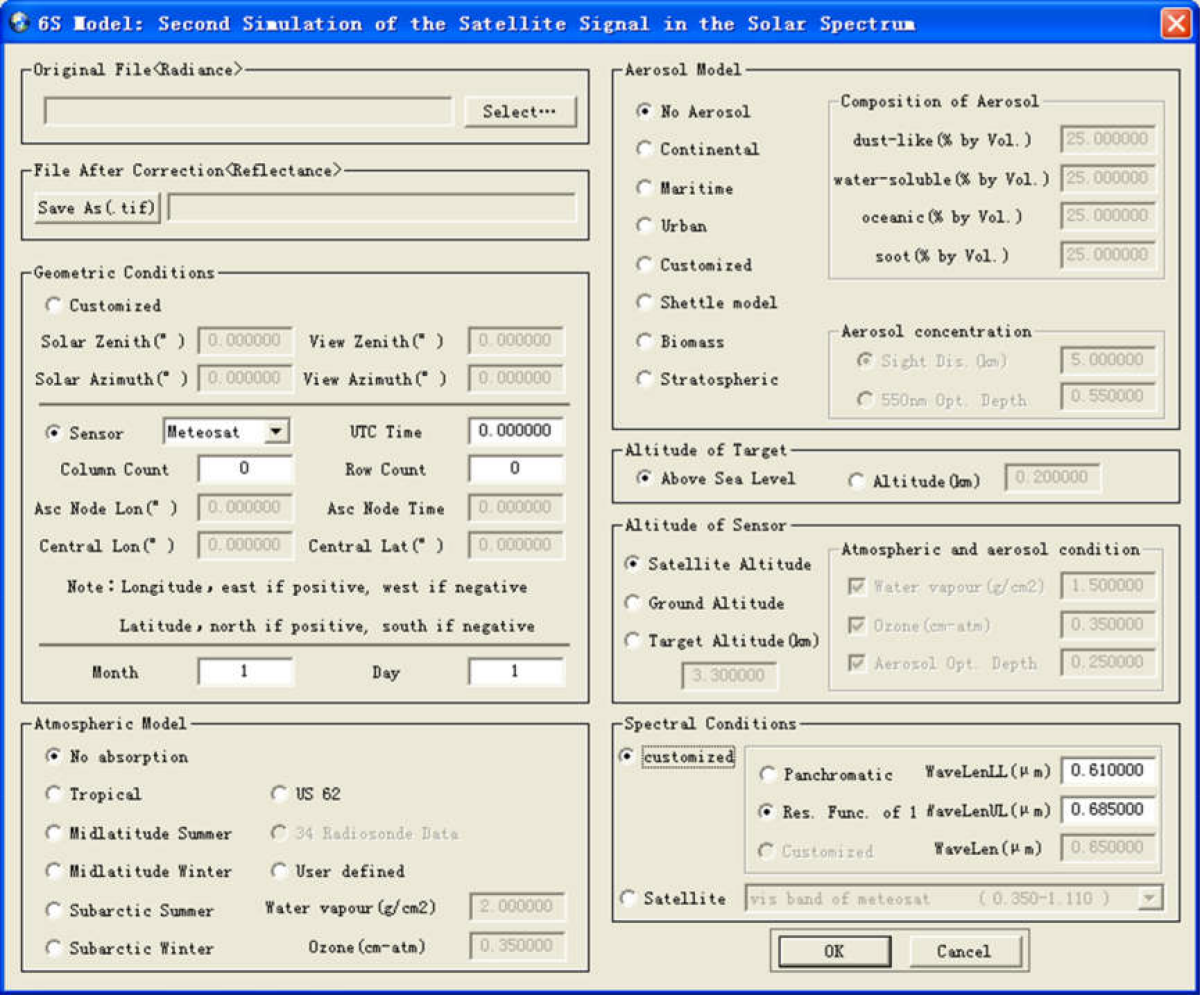
Atmospheric correction dialog.

In RSDMS, the atmospheric correction of remote sensing images are performed based on the Second Simulation of the Satellite Signal in the Solar Spectrum Model (6S Model) [38,39]. In the 6S model, the altitude of target, the non-uniform characteristics of surface and the absorption effect of gas on radiation are taken into account, and the Successive Order of Scattering algorithm is used to calculate the molecular and aerosol scattering, which enhances the accuracy of atmospheric correction. Fig 8 shows the atmospheric correction dialog.

**Fig 9:**
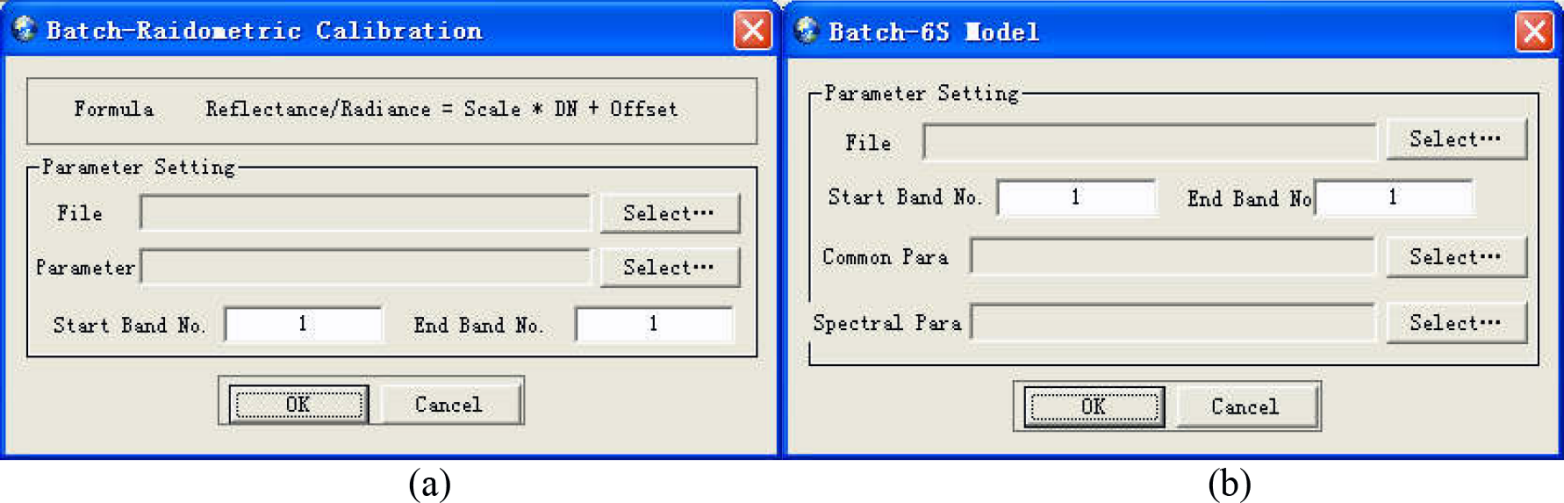
Image batch processing. (a) Batch radiometric calibration, (b) Atmospheric correction.

Considering radiometric calibration and atmospheric correction are often performed in the drought monitoring service on a large number of image pixels corresponding to the observation stations, we develop a function to batch process radiometric calibration (Fig 9A) and atmospheric correction (Fig 9B). In this function, a large number of images are batch processed based on the parameters in a prepared configuration file, which avoids repetitive manual operations.

#### 3.2.4 Drought monitoring and evaluation

The model base of RSDMS contains two types of models: internal models and external models. The former are the models implemented when the system is being developed, while the latter are the models imported to the system afterwards. The internal models can be further grouped into two categories from a functional point of view: inversion model of surface parameters, such as Normalized Difference Vegetation Index (NDVI), Albedo, Apparent Thermal Inertia (ATI) etc.; drought monitoring model, such as VCI, PDI, TVDI, etc. Table 3 shows the calculation formula and references of all internal models.

**Table 3:** Internal models of RSDMS.

Category	Index name	Formula	Reference
Inversion models of surface parameters	NDVI [40]	*NDVI* = (*ρ*_*NIR*_ − *ρ*_Re*d*_)/(*ρ*_*NIR*_ + *ρ*_Re*d*_)	Deering, 1978
	ATI [41]	*ATI* = (1 − *Albedo*)/(*T*_max_ − *T*_min_)	Tian and Yang, 1992
	NDWI [42]	*NDWI* = (*ρ*_*Green*_ − *ρ*_*NIR*_)/(*ρ*_*Green*_ + *ρ*_*NIR*_)	McFeeters, 1996
	MNDWI [43]	*MNDWI* = (*ρ*_*Green*_ – *ρ*_*MIR*_)/(*ρ*_*Green*_ + *ρ*_*MIR*_)	Xu, 2006
Drought monitoring models	VSWI [44]	*VSWI* = 100 × *NDVI*/*T*_*s*_	Carlson et al., 1994
	VCI [45]	$VCI=\frac{NDVI-NDVI_{\min}}{NDVI_{\max}-NDVI_{\min}}\times 100$	Kogan, 1995
	TVDI [23]	*TVDI* = (*T*_*s*_ − *T*_*s*,min_)/(*T*_*s*,max_ − *T*_*s*,min_)	Sandholt et al., 2002
	SIWSI [46]	$SIWSI=\frac{\rho_{SWIR}-\rho_{NIR}}{\rho_{SWIR}+\rho_{NIR}}$	Fensholt et al., 2003
	PDI [24]	PDI=1M2+1(ρRed+MρNIR)	Zhan et al., 2007
	MPDI [47]	MPDI=ρRed+MρNIR−fv(ρRed+MρNIR,v)(1−fv)M2+1	Ghulam et al., 2007a
	VCADI [47]	$VCADI=\frac{A_{NDVI}-A_{\min NDVI}}{A_{\max NDVI}-A_{\min NDVI}}$	Ghulam et al., 2007a
	SPSI [25]	$SPSI=\frac{1}{\sqrt{M^{2}+1}}(\rho_{SWIR}+M\rho_{NIR})$	Ghulam et al., 2007b
	NPDI [48]	$NPDI=\frac{1}{\sqrt{M^{2}+1}}(R_{s}+MR_{d})$	Feng et al., 2011

**Fig 10:**
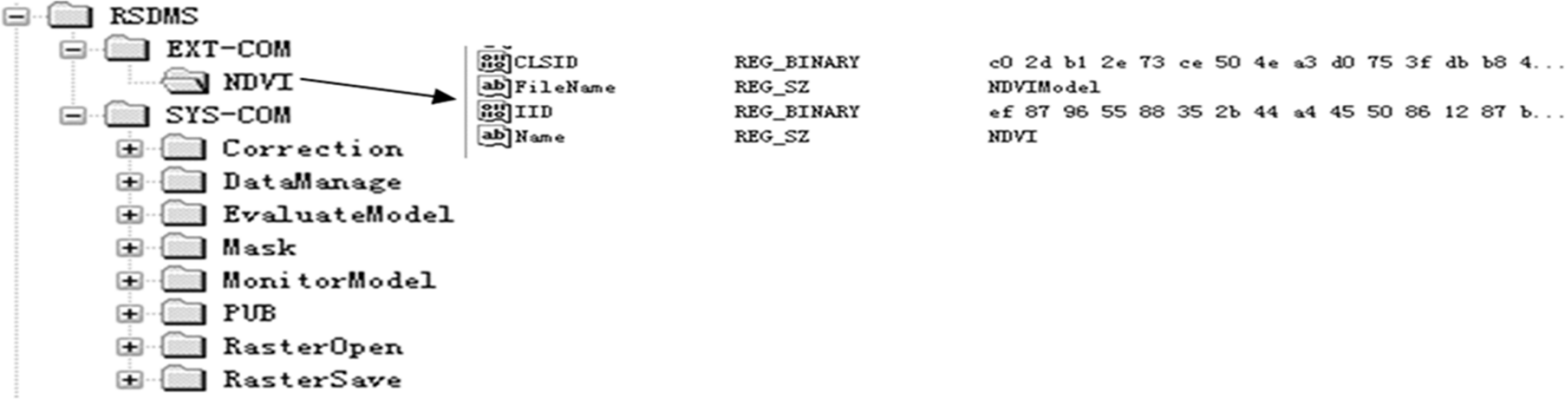
Model information of RSDMS in the registry.

**Fig 11:**
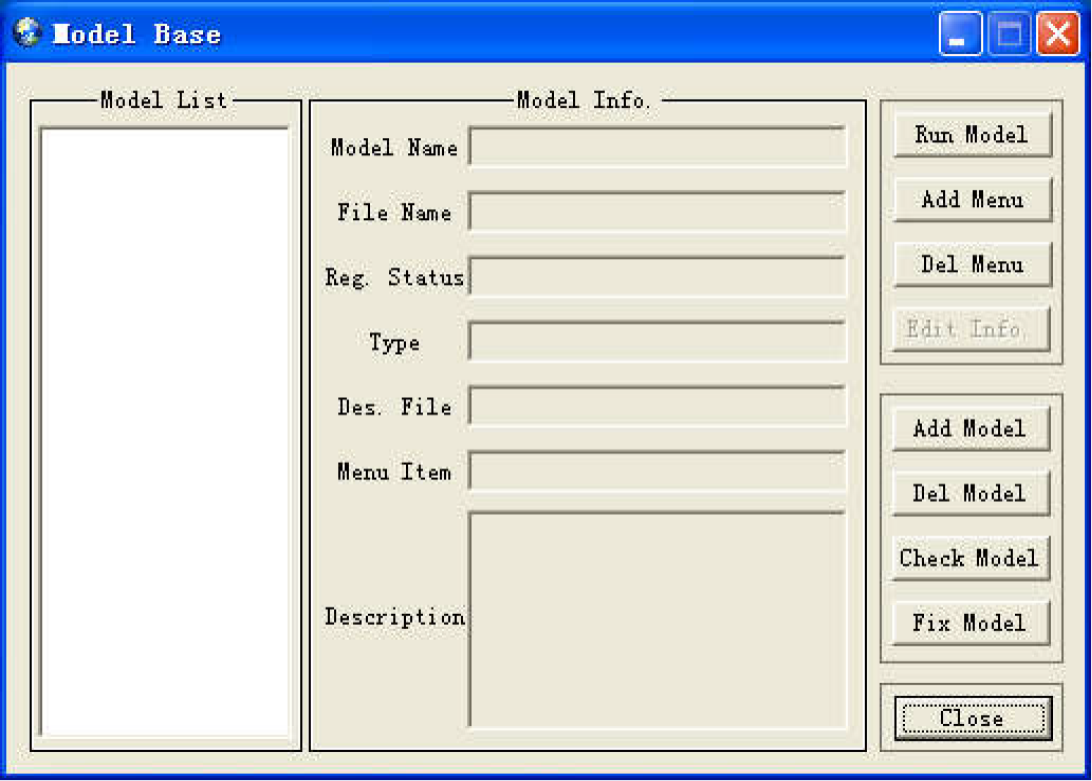
Model management dialog.

In addition to internal models, users can also use the model management function to add new models into the system according to the specified format. In order to represent the internal and external models in a unified way, we design the composition structure of a model: a model file (in the form of Dynamic Linking Library, DLL) and a description file (in the form of a binary file). Both types of models are managed by the Model Base Management Component and the registry editor in the operation system. The first thing to add a new model is to check-in through the registry editor, that is, to write the basic information (such as CLSID, IID, File Name, etc.) of the new model into the registry (Fig 10). Then the DLL file of the model is registered in RSDMS through the Model Base Management Component. The Model Base Management Component has a friendly user interface (Fig 11) with functions: add/remove, registration/unregistration, run model, description edit etc. Users can easily link an external model to RSDMS by this component.

**Fig 12:**
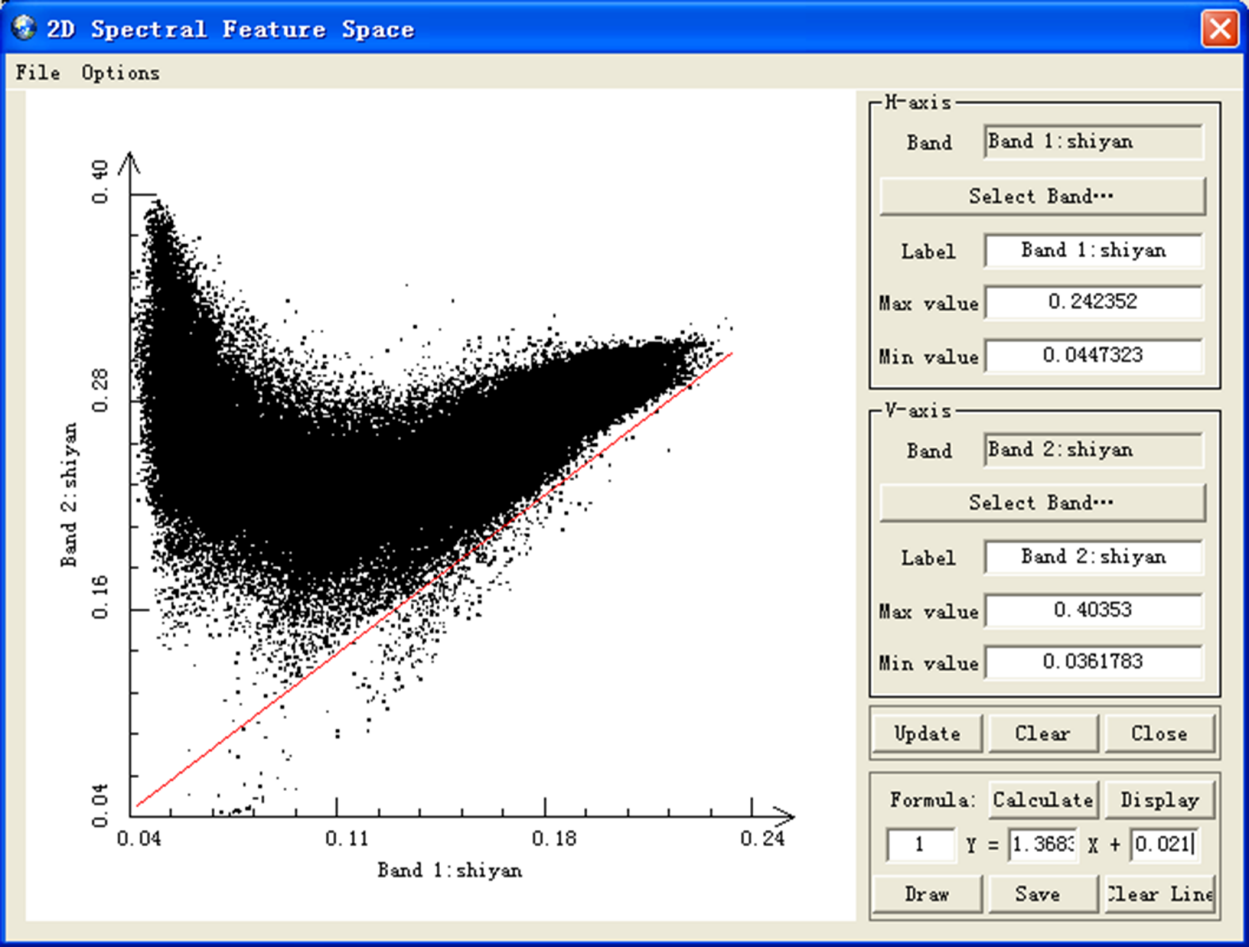
Two-dimensional spectral feature space dialog.

An important procedure of drought monitoring service is to analyze the two-dimensional spectral feature space. RSDMS provides the function of generating a scatter plot (Fig 12) which demonstrates the distribution characteristics of pixel values of any two image bands in two-dimensional feature space. Furthermore, a feature line can be obtained based on the scatter plot in both manual and automatic way. The manual way is to let users draw a feature line by the mouse operations, while the automatic way is an extension of the automated soil line identification routine proposed by Fox et al. [49]. The feature line (the red line in Fig 12) can be used as the soil line in some inversion models.

**Fig 13:**
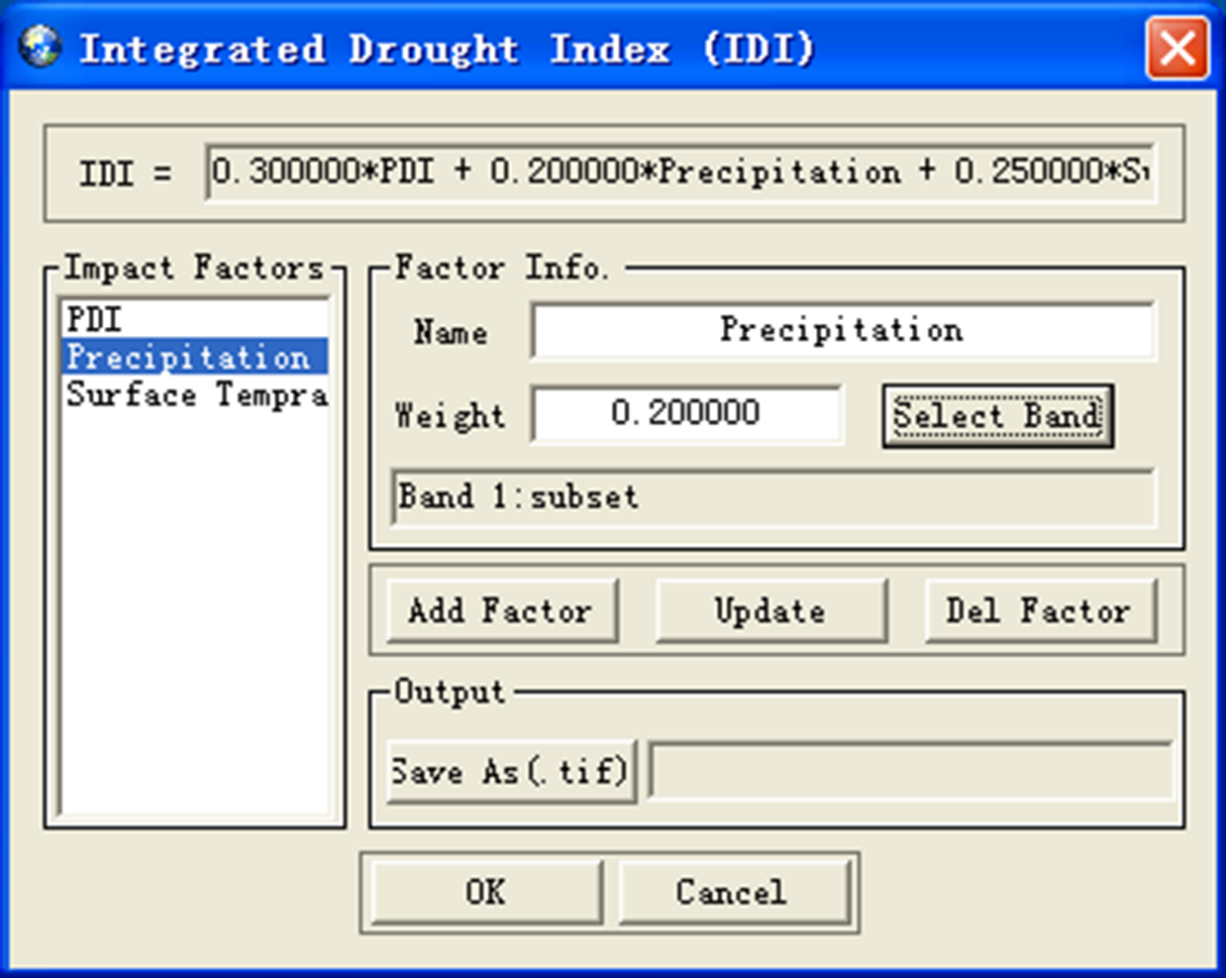
Integrated drought index dialog.

**Fig 14:**
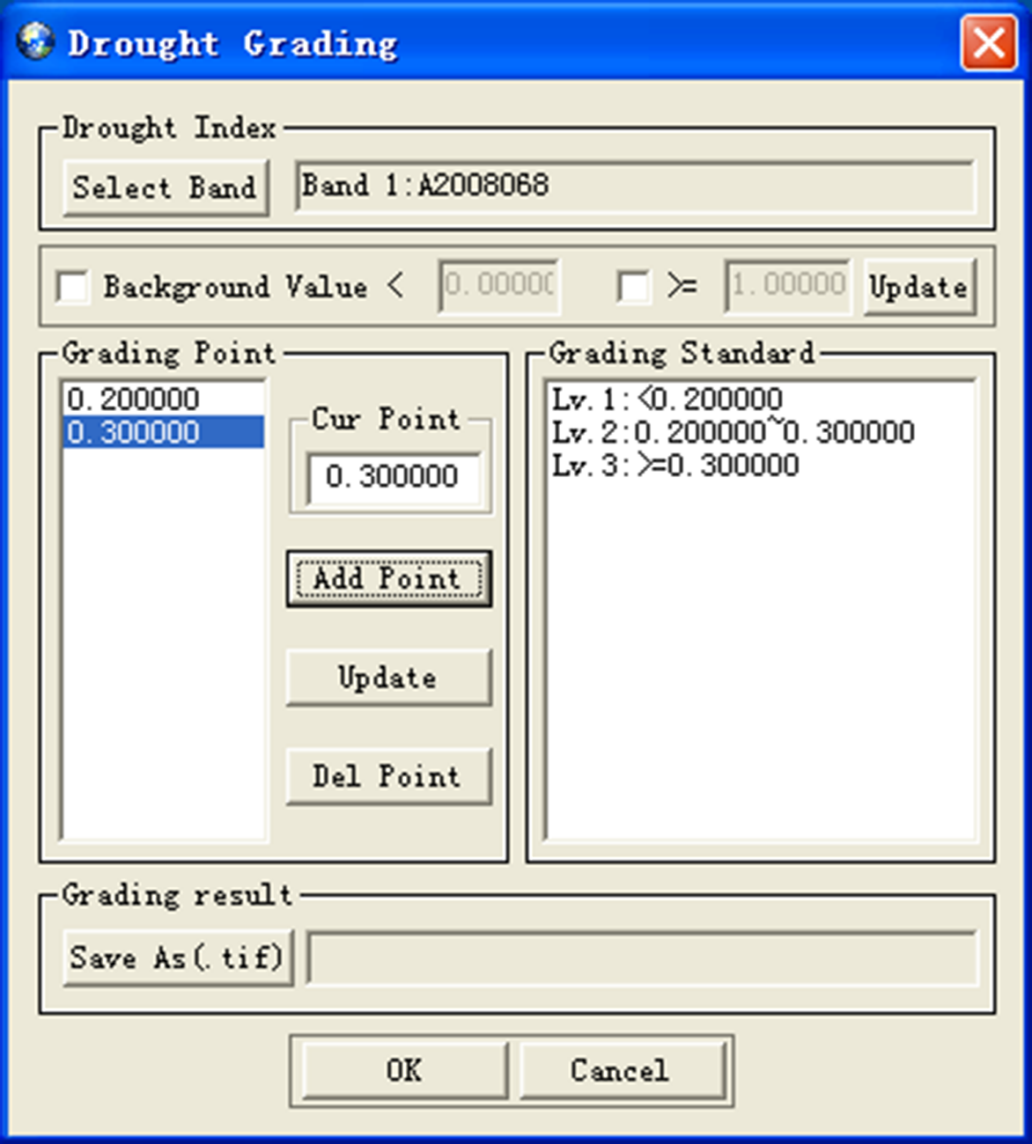
Drought rating dialog.

Aiming at evaluating the degree of drought, we develop the Integrated Drought Index (IDI) (Fig 13) which is the weighted average of drought indexes from drought monitoring models and other impact factors. The IDI takes into account the influence of various factors on regional drought and can overall reflect the degree of regional drought. Drought rating is (Fig 14) to design a drought rating standard based on IDI and remote sensing images, and display the drought region according to the grade of drought.

#### 3.2.5 Output result and mapping

**Fig 15:**
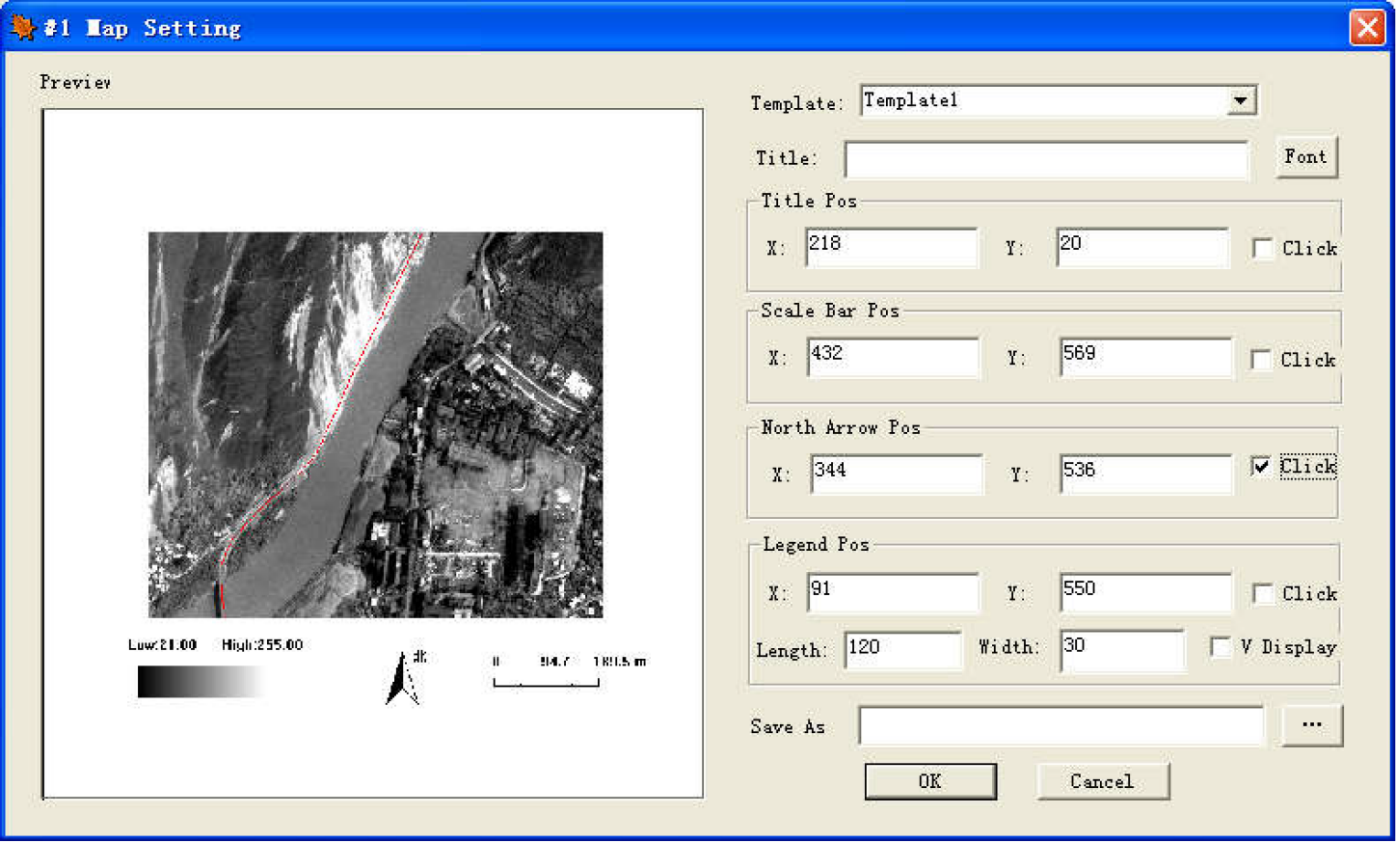
Export map dialog.

RSDMS can output the surface parameter inversion result, drought monitoring result, drought rating result in typical raster data formats. RSDMS can also provide many map layouts (Fig 15) with elements such as text annotation, scale bar, legend, north arrow, and create thematic maps by combing map elements with drought monitoring and evaluation results.

## 4. Application

To verify the practicality of RSDMS, the system is applied to drought monitoring and assessment of China’s Ningxia region.

### 4.1. Study area and data

**Fig 16:**
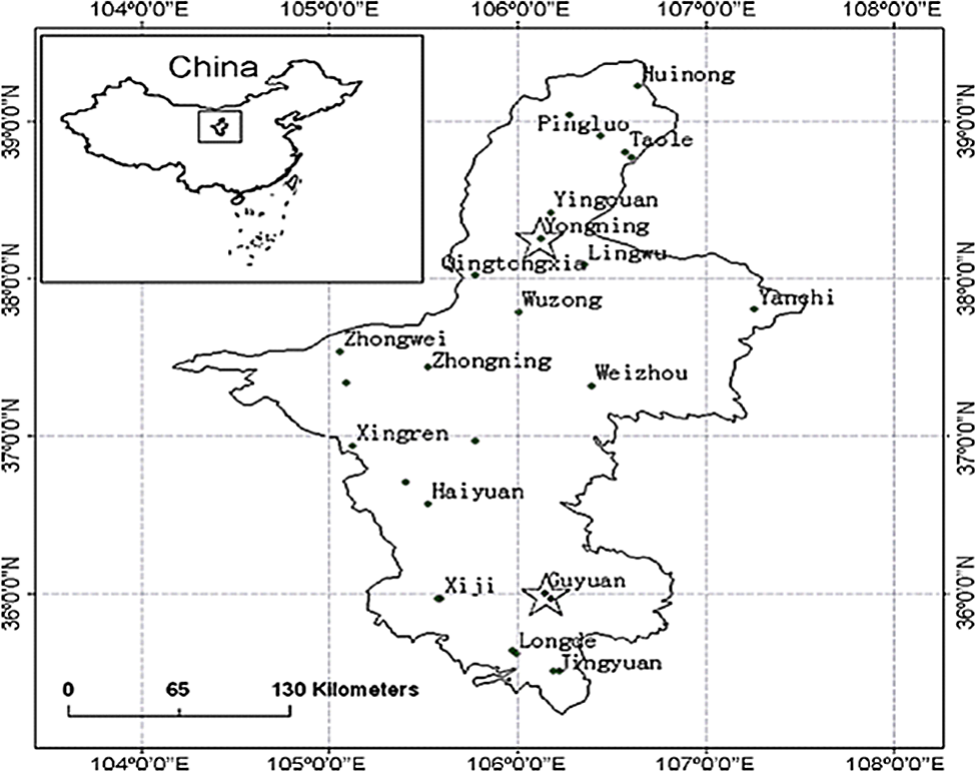
Export map dialog[36].

Ningxia Huizu Autonomous Region is located in the northwestern China, upstream of Yellow River with the geographic extent of 104°10′ E to 107°30′ E and 35°25′ N to 39°25′ N (Fig 16). The total area of Ningxia region is 51 800 km^2^ comprising typically arid and semi-arid eco-systems. Drought is a frequent disaster in this area and has serious negative effect on the local economic development. Due to the fragile eco-system, the crops in this area are often exposed to water shortage during growing seasons.

To ensure the representativeness, two typical agro-climatic sites Yongning (106°15′ E, 38°15′ N) and Guyuan (106°18′ E, 35°58′ N) were selected as the observation stations of ground data (marked as 16 pentagons in Fig 16). Yongning, a water-controlled site, is located in the Yellow River irrigation zone where crops have been fully irrigated. Guyuan is located in the southern mountainous region of Ningxia where agriculture heavily depends on rainfall. Twenty-four MOD09 products of the study area in 2009 were selected as the study data. Synchronized with the image acquisition time, we surveyed the surface parameter data of six land parcel (1km×1km) at the two observation stations. The six parcels include: two fields of interplanted wheat and corn in Yongning, two fields of winter wheat and two fields of corn in Guyuan.

The soil moistures (Volumetric Water Content, VWC) in 0–10cm soil layer in three types of farmland were measured during April to July, and they constitute a complete ground observation dataset. The soil moistures were measured using Instrument for Measuring Temperature & Moisture of Soil with±3% accuracy and 0.1% resolution made by Hash Company in the United Stated of America.

### 4.2 Drought monitoring

**Fig 17:**
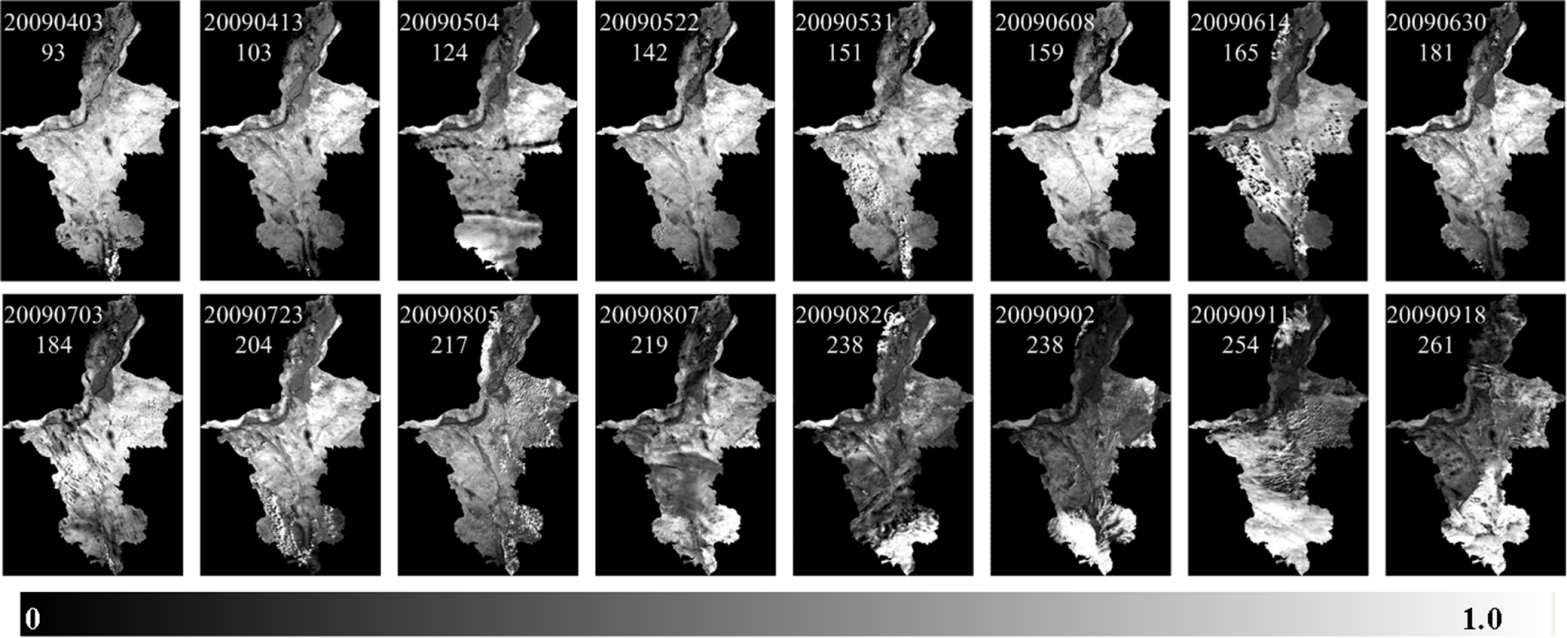
SPSI_7,2_ result over the Ningxia Plain [36].

We chose PDI, MPDI, SPSI and SIWSI to monitor Ningxia drought conditions. These four vegetation indices are good methods to monitor soil water content [50,51]. We imported MOD09 images into RSDMS and conducted all preprocessing procedures including image subset, radiometric calibration, atmospheric correction and projection transformation. Then the first band (620–670nm), second band (841–876 nm) and the seventh band (2105–2155 nm) of images were chosen to construct the RED-NIR and NIR-SWIR feature spaces for the four vegetation Indices, and further the RED-NIR and NIR-SWIR base lines were acquired using the automatic feature line extraction function of RSDMS. The results of spatial distribution of drought over Ningxia plain with four vegetation indices were similar. Taking SPSI index as an example, Fig 17 shows the spatial distribution of drought over Ningxia plain on different dates in 2009.

**Fig 18:**
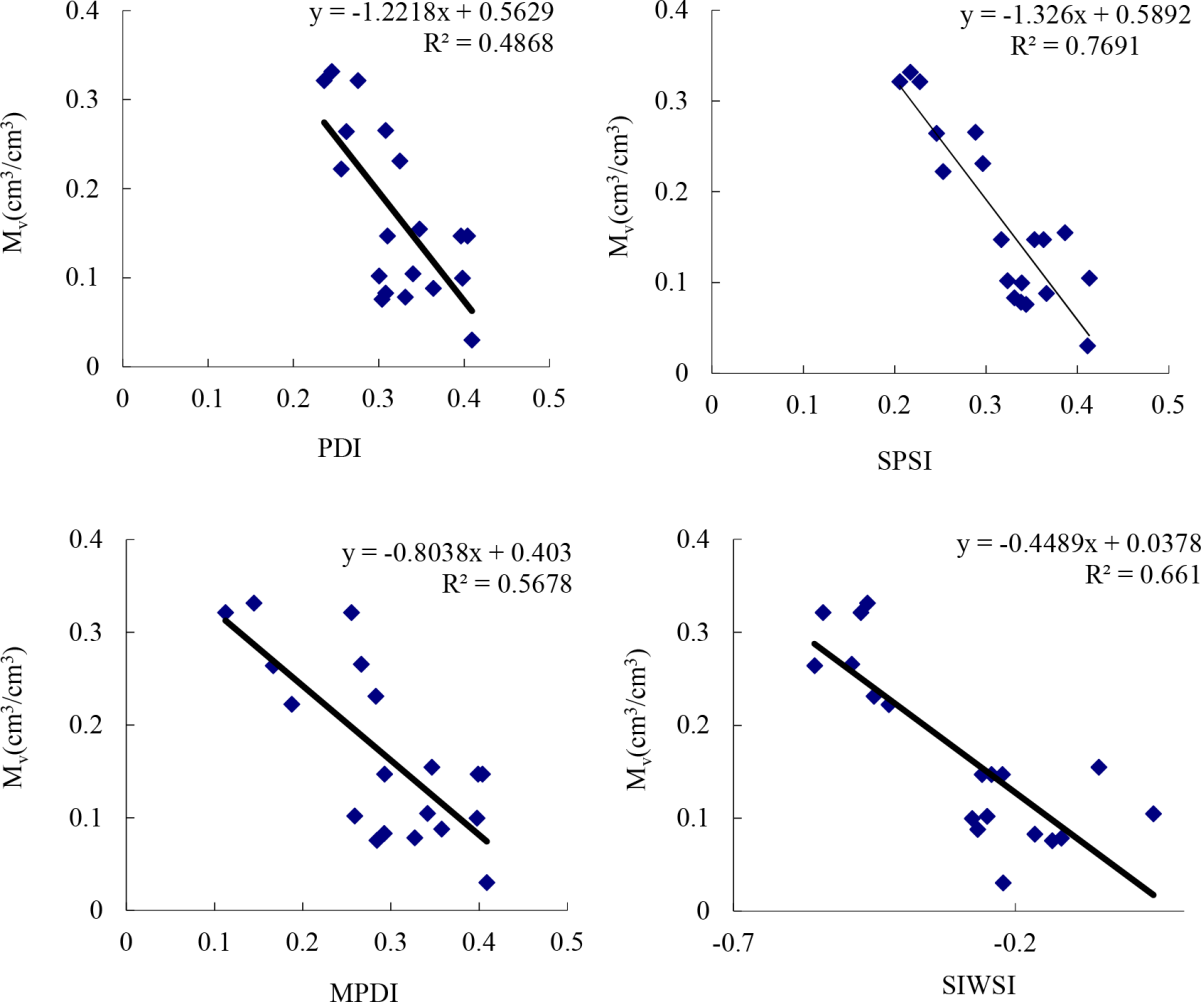
Inversion models for soil and leaf water content.

The vegetation indices values of image pixels corresponding to the two observation sites can be obtained by the information query tool. Part of the ground-measured surface data were used for established an inversion model. The linear regression was conducted on the ground-measured data and their corresponding vegetation Indices values to obtain the inversion models for the water content of soil (Fig 18). All four regression functions obtain a small p-value (P < 0.01), which indicates that there is a significant correlation between indices and the in-situ measurements. In addition, we can see that SPSI is the best vegetation index for monitoring drought in Ningxia.

**Fig 19:**
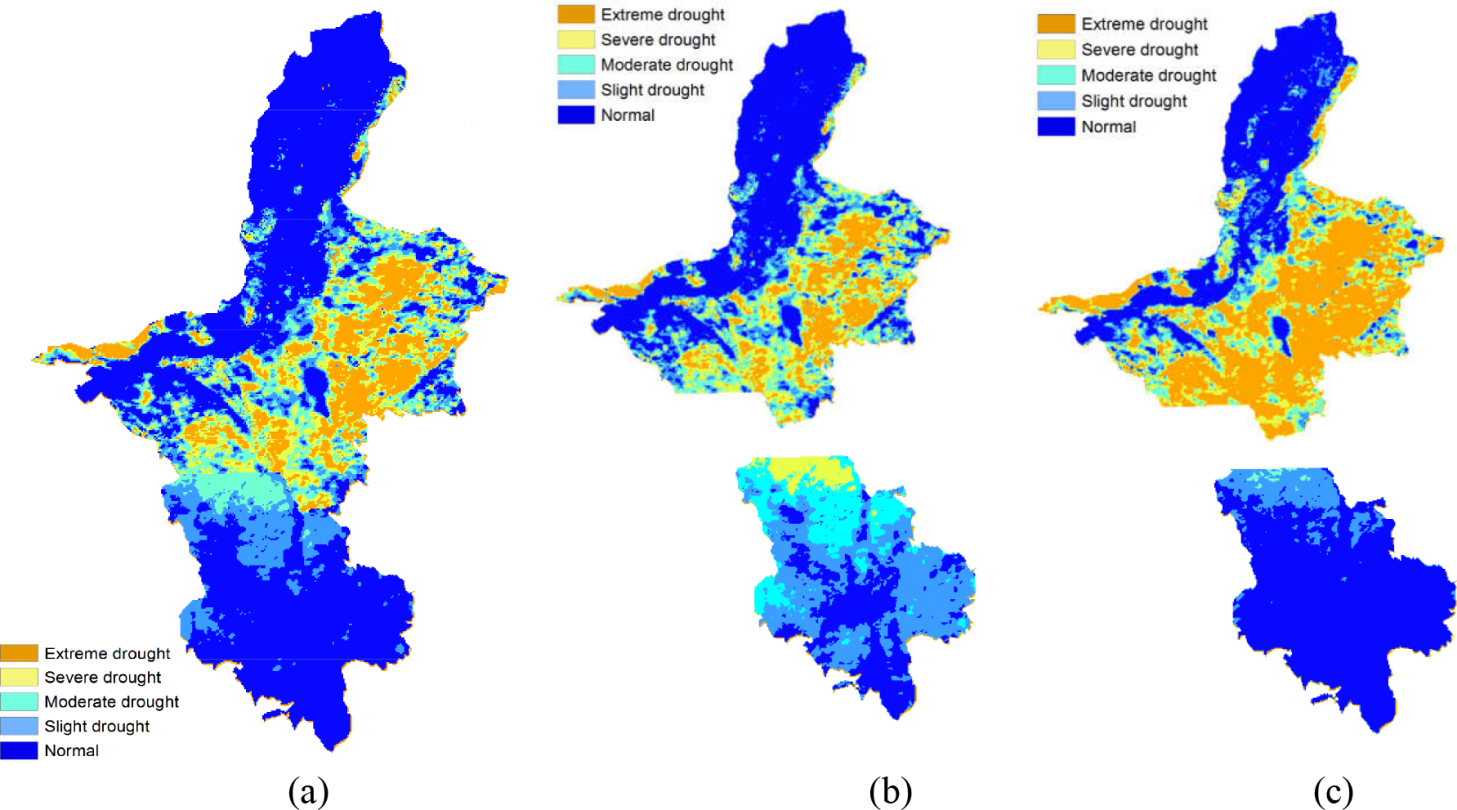
Two types of drought rating result of Ningxia. (a) Meteorological drought rating, (b) Agricultural drought rating of wheat, (c) Agricultural drought rating of corn [54].

We then took the dataset in June as a sample, and used the drought rating feature to evaluate the risk level of drought disaster in Ningxia. First, we set the rating threshold values according to China’s meteorological drought rating standard [52] and obtain the meteorological drought rating result in Fig 19A. Second, considering the crops have different levels of water requirements in different growing periods [53], we further set the rating threshold values based on crop growing periods and generate the agricultural drought rating result of wheat and corn as shown in Fig 19B and 19C.

**Fig 20:**
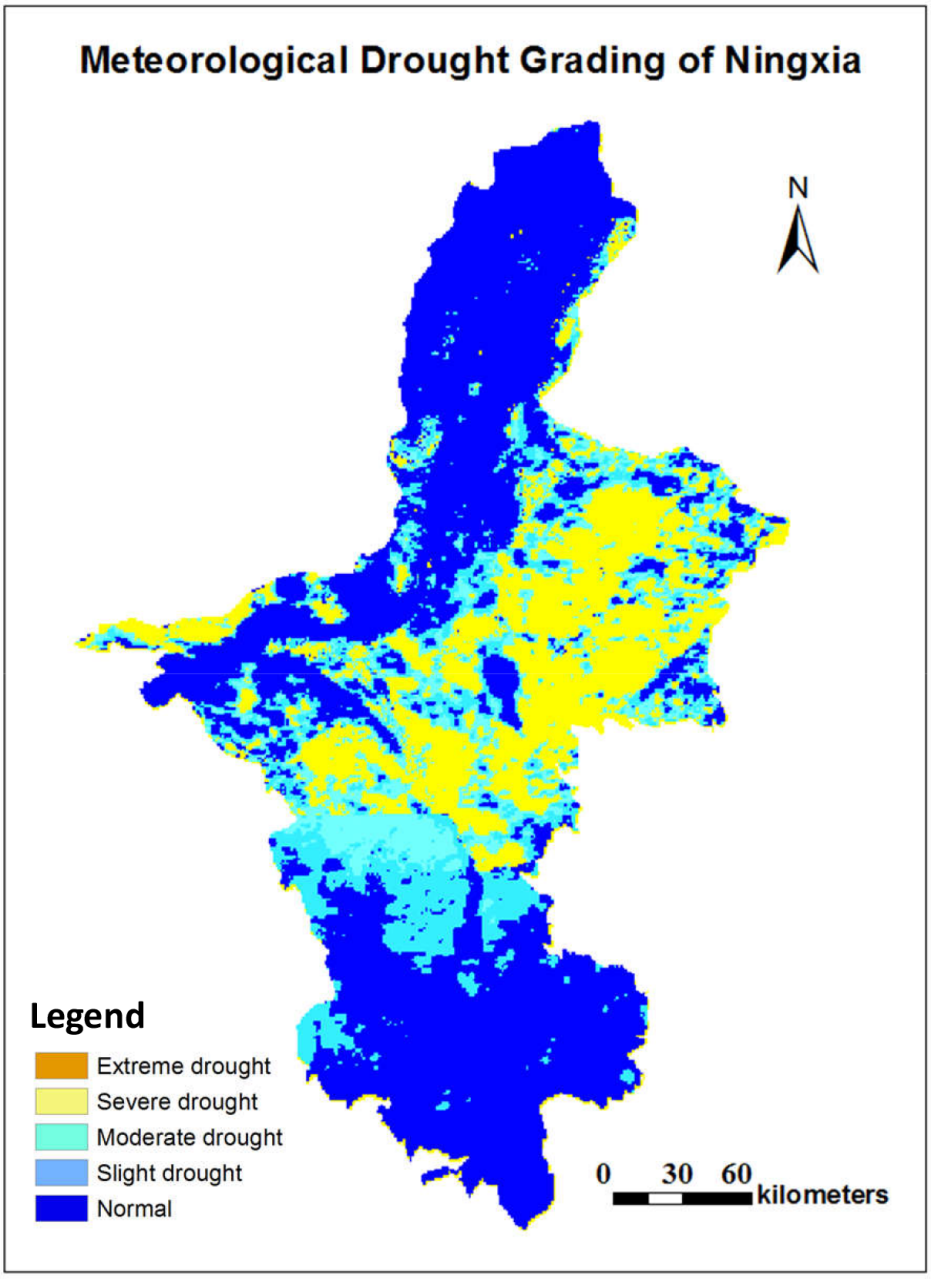
Drought evaluation mapping result[53].

Due to the influence of terrain and climate, the growing period of crops in the middle north region is significantly different from that in the southern region of Ningxia, therefore, the drought status in these two regions are evaluated separately. It can be seen from Fig 19 that as for the wheat in the middle north region (the top part in Fig 19B), the meteorological drought rating result is basically consistent with the agricultural drought rating result, and this is because the wheat in this region is at the late growth stage. For the wheat in the south region (the bottom part in Fig 19B) and the corn in the middle north region (the top part in Fig 19C), the agricultural drought grade is mostly higher than the meteorological drought grade since the crops are at the vigorous stage and have a large water demand. For the corn in the south region (the bottom part in Fig 19C), the agricultural drought grade is lower than the meteorological drought grade since the corn is at the seedling stage and requires a small amount of water. Fig 19 also shows that the soil water content imposes different levels of stress on different crops in the same region, and thus RSDMS can implement agricultural drought evaluation based on the growth periods of crops and provide practical instructions on crop planting. After the final result was obtained, the mapping module of RSDMS can insert necessary map elements such as the map title, north arrow, scale bar, legend etc. to output the result as shown in Fig 20.

## 5. Conclusion

There is an urgent need for a powerful and extendable remote sensing drought monitoring system in advanced research and professional work. The current systems such as GADMFS and GIDMaPS focus mainly on disseminating near real-time global drought monitoring information through web service technology or providing comprehensive global drought information by integrating multiple drought indicators. However, it is difficult for these systems to handle the continuous advent of drought indicators and the need of advanced data analysis. In order to fix this gap, we developed a component based system for agricultural drought monitoring by remote sensing, namely RSDMS. From the functional point of view, RSDMS is both a general image processing system and a professional drought monitoring system. It not only incorporates a variety of widely-used drought monitoring and evaluation models, but also achieves data preprocessing and management, image analysis etc. For instance, RSDMS can support all mainstream vector and raster data formats, especially remotely sensed images. Besides, RSDMS can handle large image datasets and provide a friendly user interface. More importantly, the most attractive feature of RSDMS is the strong flexibility owing to the flexible component-based architecture. On one hand, both the Functionality Component Library and the Monitoring and Evaluation Model Base are scalable. New functionalities and professional models can be easily added into the existing platform and managed well. On the other hand, the applications of RSDMS are not limited to drought monitoring. If RSDMS was embedded with new models in a new field such as the water quality parameter inversion models, it would become a new support platform.

Future work would be focused on the integration of RSDMS and the web-based drought information publishing system for accomplishing fast modeling and information publishing as well. Improving the efficiency and convenience of the workflows of professional modeling according to the practical regulations of drought monitoring organizations would be another important work to consider.
